# The Feasibility of Canine Rabies Elimination in Africa: Dispelling Doubts with Data

**DOI:** 10.1371/journal.pntd.0000626

**Published:** 2010-02-23

**Authors:** Tiziana Lembo, Katie Hampson, Magai T. Kaare, Eblate Ernest, Darryn Knobel, Rudovick R. Kazwala, Daniel T. Haydon, Sarah Cleaveland

**Affiliations:** 1 Boyd Orr Centre for Population and Ecosystem Health, University of Glasgow, Glasgow, United Kingdom; 2 Davee Center for Epidemiology and Endocrinology, Lincoln Park Zoo, Chicago, Illinois, United States of America; 3 Department of Animal and Plant Sciences, University of Sheffield, Western Bank, Sheffield, United Kingdom; 4 Serengeti Carnivore Viral Transmission Dynamics Project, Tanzania Wildlife Research Institute, Arusha, Tanzania; 5 Sokoine University of Agriculture, Department of Veterinary Medicine and Public Health, Morogoro, Tanzania; Centers for Disease Control and Prevention, United States of America

## Abstract

**Background:**

Canine rabies causes many thousands of human deaths every year in Africa, and continues to increase throughout much of the continent.

**Methodology/Principal Findings:**

This paper identifies four common reasons given for the lack of effective canine rabies control in Africa: (a) a low priority given for disease control as a result of lack of awareness of the rabies burden; (b) epidemiological constraints such as uncertainties about the required levels of vaccination coverage and the possibility of sustained cycles of infection in wildlife; (c) operational constraints including accessibility of dogs for vaccination and insufficient knowledge of dog population sizes for planning of vaccination campaigns; and (d) limited resources for implementation of rabies surveillance and control. We address each of these issues in turn, presenting data from field studies and modelling approaches used in Tanzania, including burden of disease evaluations, detailed epidemiological studies, operational data from vaccination campaigns in different demographic and ecological settings, and economic analyses of the cost-effectiveness of dog vaccination for human rabies prevention.

**Conclusions/Significance:**

We conclude that there are no insurmountable problems to canine rabies control in most of Africa; that elimination of canine rabies is epidemiologically and practically feasible through mass vaccination of domestic dogs; and that domestic dog vaccination provides a cost-effective approach to the prevention and elimination of human rabies deaths.

## Author Summary

Elimination of canine rabies has been achieved in some parts of the world, but the disease still kills many thousands of people each year in Africa. Here we counter common arguments given for the lack of effective canine rabies control in Africa presenting detailed data from a range of settings. We conclude that (1) rabies substantially affects public and animal health sectors, hence regional and national priorities for control ought to be higher, (2) for practical purposes domestic dogs are the sole maintenance hosts and main source of infection for humans throughout most of Africa and Asia and sufficient levels of vaccination coverage in domestic dog populations should lead to elimination of canine rabies in most areas, (3) the vast majority of domestic dog populations across sub-Saharan Africa are accessible for vaccination with community sensitization being of paramount importance for the success of these programs, (4) improved local capacity in rabies surveillance and diagnostics will help evaluate the impact of control and elimination efforts, and (5) sustainable resources for effective dog vaccination campaigns are likely to be available through the development of intersectoral financing schemes involving both medical and veterinary sectors.

## Introduction

Rabies is a viral zoonosis caused by negative-stranded RNA viruses from the *Lyssavirus* genus. Genetic variants of the genotype 1 Lyssavirus (the cause of classical rabies) are maintained in different parts of the world by different reservoir hosts within ‘host-adaptive landscapes’ [1]. Although rabies can infect and be transmitted by a wide range of mammals, reservoirs comprise only mammalian species within the Orders *Carnivora* (e.g. dogs, raccoons, skunks, foxes, jackals) and *Chiroptera* (bats). From the perspective of human rabies, the vast majority of human cases (>90%) result from the bites of rabid domestic dogs [2] and occur in regions where domestic dogs are the principal maintenance host [3].

Over the past three decades, there have been marked differences in efforts to control canine rabies. Recent successes have been demonstrated in many parts of central and South America, where canine rabies has been brought under control through large-scale, synchronized mass dog vaccination campaigns [4]. As a result, not only has dog rabies declined, but human rabies deaths have also been eliminated, or cases remain highly localized [5]. The contrast with the situation in Africa and Asia is striking; here, the incidence of dog rabies and human rabies deaths continue to escalate, and new outbreaks have been occurring in areas previously free of the disease (e.g. the islands of Flores and Bali in Indonesia – [6]; http://wwwn.cdc.gov/travel/contentRabiesBaliIndonesia2008.aspx).

In this paper, we identify four major reasons commonly given for the lack of effective domestic dog rabies control including (1) low prioritisation, (2) epidemiological constraints, (3) operational constraints and (4) lack of resources (Table 1), focussing on the situation in Africa. We address each of these issues in turn, using outputs from modelling approaches and data from field studies to demonstrate that there are no insurmountable logistic, practical, epidemiological, ecological or economic obstacles. As a result, we conclude that the elimination of canine rabies is a feasible objective for much of Africa and there should be no reasons for further delay in preventing the unnecessary tragedy of human rabies deaths.

**Table 1:** Reasons commonly given for the lack of effective dog rabies control.

Reason	Explanation	Oral evidence	Published evidence
LOW PRIORITISATION	Lack of accurate data on the disease burden and low recognition among public health practitioners and policy makers; lack of inclusion of rabies in global surveys of disease burden; only recent recognition of rabies as a neglected tropical disease; statements of rabies as an ‘insignificant human disease’	Ministries of Health; statements by doctors and health workers; WHO (up until 2007)	I-VI*
EPIDEMIOLOGICAL CONSTRAINTS	Abundance of wild animals and uncertainties about the required levels of vaccination coverage	SEARG meetings, scientific meetings, national veterinary meetings; statements from district veterinary officers and local communities; draft rabies control policies	VII-XIX
OPERATIONAL CONSTRAINTS	Perception of existence of many inaccessible stray/ownerless dogs	SEARG meetings, inter-ministerial meetings, national veterinary meetings; statements from district veterinary and medical officers, and livestock officers; draft rabies control policies; international organizations	XX-XXVIII
	Owners unwilling or unable to bring dogs for vaccination	SEARG meetings, inter-ministerial meetings, national veterinary meetings, scientific meetings; statements by veterinary and livestock officers	XXIX,XXX
	Insufficient knowledge of dog population size and ecology	SEARG meetings, inter-ministerial meetings, scientific meetings; statements from veterinary and livestock officers and wildlife authorities; draft rabies control policies; international organizations	XIV,XXIV,XXXI
LACK OF RESOURCES	Weak surveillance and diagnostic capacity	SEARG meetings, inter-ministerial meetings; international and national reference laboratories; international organizations	VI,XXIII,XXIV,XXXII-XXXVIII
	Insufficient resources available to veterinary services	SEARG meetings, inter-ministerial meetings, scientific meetings, national veterinary meetings; statements from politicians, veterinary authorities, local communities, wildlife authorities; international organizations; media	XXVI,XXXIV,XXXVII,XXXIX,XL-XLIII

SEARG = Southern and Eastern Africa Rabies Group.

*Including indirect evidence (e.g. absence of any mention of rabies in published literature indicating lack of priority). See Appendix S1 for references.

## Methods

This paper compiles previously published data (see references below) and additional analyses of those data, but we present a brief summary of the data collection methods below.

Hospital records of animal-bite injuries compiled from northwest Tanzania were used as primary data sources. These data informed a probability decision tree model for a national disease burden evaluation [7], which has since been adapted for global estimates of human rabies deaths and Disability-Adjusted Life Years (DALYs) lost due to rabies [3], a standardized measure for assessing disease burden [8],[9]. Hospital records were also used to initiate contact tracing studies [10]–[12], whereby bite-victims were interviewed to obtain more detail on the source and severity of exposure and actions taken, allowing subsequent interviews with other affected individuals (not documented in hospital records) including owners of implicated animals. Statistical techniques applied to these data for estimating epidemiological parameters and inferring transmission links are described elsewhere [10],[12].

Rabies monitoring operations including passive and active surveillance involving veterinarians, village livestock field officers, paravets, rangers and scientists were used to collect samples from carcasses (domestic dogs and wildlife whenever found), which were subsequently tested and viral isolates were sequenced [10], [13]–[16], with results being used to inform estimates of rabies-recognition probabilities [7] and for phylogenetic analyses [10],[16]. Operational research on domestic dog vaccination strategies was carried out in a variety of settings [14],[17]. Household interviews were also used for socio-economic surveys and to evaluate human:domestic dog ratios, levels of vaccination coverage achieved and reasons for not bringing animals to vaccination stations [17],[18].

The study was approved by the Tanzania Commission for Science and Technology with ethical review from the National Institute for Medical Research (NIMR). This retrospective study involved collection of interview data only, without clinical intervention or sampling, therefore we considered that informed verbal consent was appropriate and this was approved by NIMR. Permission to conduct interviews was obtained from district officials, village and sub-village leaders in all study locations. At each household visited, the head of the household was informed about the purpose of the study and interviews were conducted with verbal consent from both the head of the household and the bite victim (documented in a spreadsheet). Approval for animal work was obtained from the Institutional Animal Care and Use Committee (IACUC permit #0107A04903).

## Results/Discussion

### (a) There is not enough evidence to define rabies control as a priority

A principal factor contributing to a low prioritization of rabies control has been the lack of information about the burden and impact of the disease [19],[20]. Data on human rabies deaths, submitted from Ministries of Health to the World Health Organization (WHO), are published in the annual World Surveys of Rabies and through the WHO Rabnet site (www.who.int/rabies/rabnet/en). For the WHO African region (AFRO) comprising 37 countries, these surveys report an average of 162 human deaths per year between 1988 and 2006. It is therefore unsurprising that for national and international policy-makers, rabies pails into insignificance in comparison with other major disease problems.

This perceived lack of significance of human rabies is reflected in the absence of any mention of rabies in either of the two published Global Burden of Disease Surveys [21],[22], which assessed more than 100 major diseases. These surveys adopted the metric of the DALY which is widely used as the principal tool for providing consistent, comparative information on disease burden for policy-making. Until recently no estimates of the DALY burden were available for rabies.

Official data on human rabies deaths submitted to WHO from Africa are widely recognized to greatly under-estimate the true incidence of disease. The reasons for this are manifold: (1) rabies victims are often too ill to travel to hospital or die before arrival, (2) families recognize the futility of medical treatment for rabies, (3) patients are considered to be the victims of bewitchment rather than disease, (4) clinically recognized cases at hospitals may go unreported to central authorities, and (5) misdiagnosis is not uncommon. The problems of misdiagnosis were highlighted by a study of childhood encephalitis in Malawi, in which 3/26 (11.5%) cases initially diagnosed as cerebral malaria were confirmed as rabies through post-mortem tests [23].

Several recent studies have contributed information that consistently demonstrates that the burden of canine rabies is not insubstantial.

#### Human rabies deaths

Estimates of human rabies cases from modeling approaches, using the incidence of dog-bite injuries and availability of rabies post-exposure prophylaxis (PEP), indicate that incidence in Africa is about 100 times higher than officially reported, with ∼24,000 deaths in Africa each year [3],[7]. Consistent figures have subsequently been generated from detailed contact-tracing data: in rural Tanzanian communities with sporadic availability of PEP (a typical scenario in developing countries), human rabies deaths occur at an incidence of ∼1–5 cases/100,000/year (equivalent to 380–1,900 deaths per year for Tanzania) [11]. Similarly, a multi-centric study from India reported 18,500 human rabies deaths per year [24], consistent with model outputs of 19,700 deaths for India [3].

**Figure 1:**
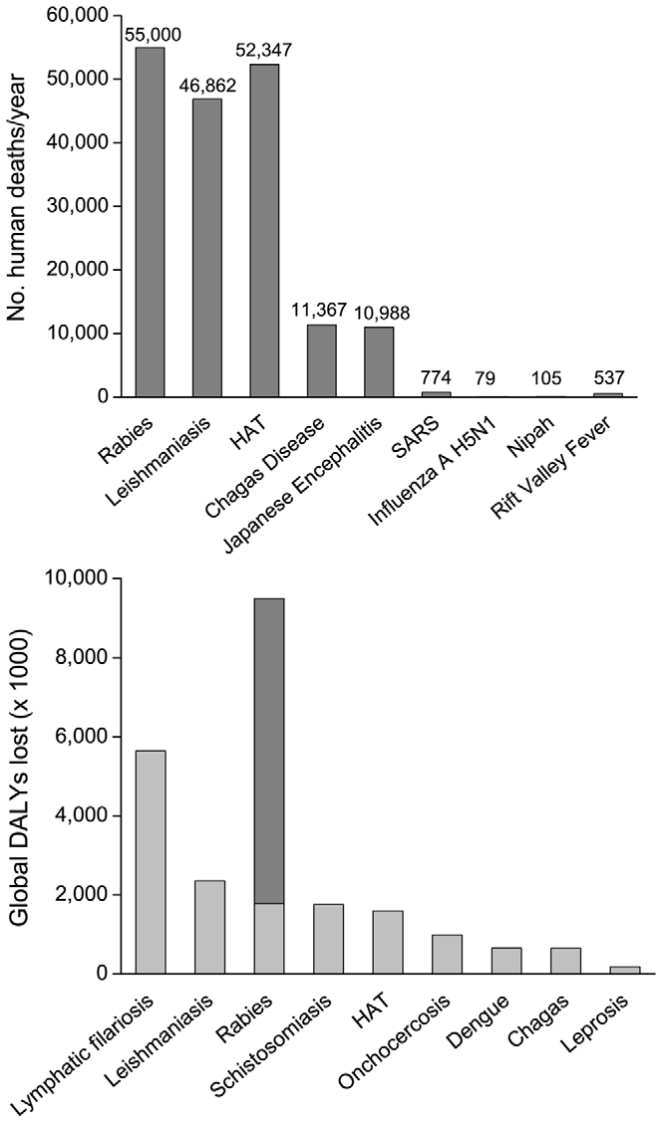
Annual human deaths for a range of zoonoses and global disability-adjusted life years (DALYs) scores for neglected zoonoses. Top figure - Numbers of human deaths per year for rabies compared with peak annual deaths from selected epidemic zoonoses (Severe Acute Respiratory Syndrome, SARS, 2003; H5N1, 2006; Nipah, 1999; and Rift Valley Fever 2007). Data sources: Rabies (LVII), Leishmaniasis, Human African Trypanosomiasis (HAT), Chagas Disease and Japanese Encephalitis (LVIII), SARS (LIX), Influenza A H5N1 (LX), Nipah (LXI), Rift Valley Fever (LXII,LXIII). See Appendix S1 for references. Bottom figure - Global DALY scores for neglected tropical diseases reported in LXIV and LVII and also assuming no post-exposure treatment (dark grey). See Appendix S1 for references.

A crude comparison of annual human deaths for a range of zoonotic diseases is shown in Figure 1 (top). While diseases such as Severe Acute Respiratory Syndrome (SARS), Rift Valley Fever and highly pathogenic avian influenza cause major concerns as a result of pandemic potential and economic losses, these figures provide a salutary reminder of the recurrent annual mortality of rabies and other neglected zoonoses, such as leishmaniasis and Human African Trypanosomiasis (HAT). Decision-tree models applied to data from East Africa and globally indicate that the DALY burden for rabies exceeds that of most other neglected zoonotic diseases (Figure 1 - bottom) [3],[25],[26].

#### Human animal-bite injuries and morbidity

Most of the rabies DALY burden is attributed to deaths, rather than morbidity because of the short duration of clinical disease. The DALY burden for rabies is particularly high, because most deaths occur in children and therefore a greater number of years of life are lost [25],[27]. DALY estimates incorporate non-rabies mortality and morbidity in terms of adverse reactions to nerve-tissue vaccines (NTVs) [3], which are still widely used in some developing countries such as Ethiopia, however rabies also causes substantial ‘morbidity’ as a direct result of injuries inflicted by rabid animals, and this is not included in DALY estimates.

Contact-tracing studies suggest an incidence as high as 140/100,000 bites by suspected rabid animals in rural communities of Tanzania [11]. Thus, for every human rabies death there are typically more than ten other rabid animal-bite victims who do not develop signs of rabies, because they obtain PEP (Figure 1 - bottom) or are simply fortunate to remain healthy. The severity of wounds has not yet been quantified, but case-history interviews suggest that injuries often involve multiple, penetrating wounds that require medical treatment.

#### Economic burden

The major component of the economic burden of rabies relates to high costs of PEP, which impacts both government and household budgets. With the phasing out of NTVs, many countries spend millions of dollars importing supplies of tissue-culture vaccine (∼$196 million USD pa [3]).

**Figure 2:**
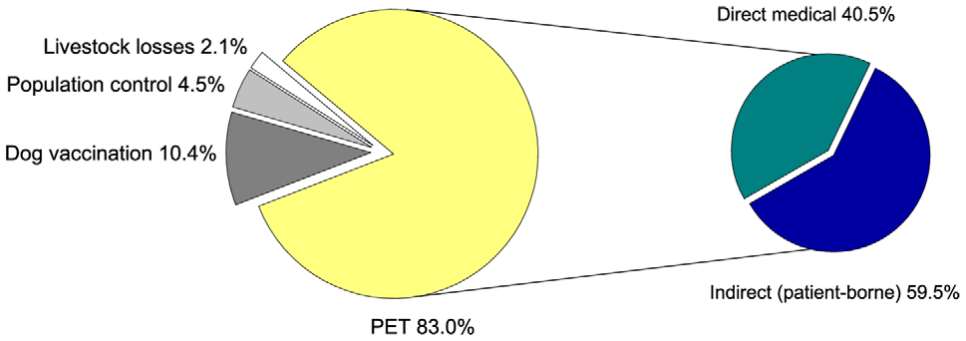
Economic burden of canine rabies (data source: LVII in Appendix S1). PET, Post-exposure treatment.

At the household level, costs of PEP arise directly from anti-rabies vaccines and from high indirect (patient-borne) costs associated with travel (particularly given the requirement of multiple hospital visits), medical fees and income loss [3],[28]. Indirect losses, represent >50% of total costs (Figure 2). Total costs have been estimated conservatively at $40 US per treatment in Africa and $49 US in Asia accounting respectively for 5.8% and 3.9% of annual per capita gross national income [3]. Poor households face difficulties raising funds which results in considerable financial hardship and substantial delays in PEP delivery [11],[28]. Shortages of PEP, which are frequent in much of Africa, further increase costs as bite victims are forced to travel to multiple centres to obtain treatment, also resulting in risky delays [11].

Additional economic losses relate to livestock losses derived from an incidence of 5 deaths/100,000 cattle estimated to cost $12.3 million annually in Africa and Asia [3]. However, substantially higher incidence has been recorded in Tanzania, with 12–25 cases/100,000 cattle reported annually in rural communities (Hampson, unpublished).

Canine rabies introduced from sympatric domestic dog populations is also recognized as a major threat to endangered African wild dogs (*Lycaon pictus*) and Ethiopian wolves (*Canis simensis*) [29]–[32]. Potential losses of tourism revenue may be substantial; African wild dogs are a major attraction in South Africa National Parks with the value of a single pack estimated at $9,000 per year [33] and Ethiopian wolves are a flagship species for the Bale Mountains National Park.

#### Psychological impact

An important, but often under-appreciated component of disease burden is the psychological impact on bite-victims and their families. In rural Tanzania, >87% of households with dog bite victims feared a bite from a suspected rabid animal more than malaria [28] because malaria can be treated whereas clinical rabies is invariably fatal and malaria treatment is generally affordable and available locally in comparison to PEP. When human rabies cases occur, the horrifying symptoms and invariably fatal outcome result in substantial trauma for families, communities and health care workers [34].

### (b) Epidemiological constraints

Increasing incidence of rabies in Africa has prompted concerns that the epidemiology of the disease may be more complex, involving abundant wildlife carnivores that may sustain infection cycles [13], [35]–[38]. There is also uncertainty about the level of vaccination coverage needed to control rabies particularly in rapidly growing domestic dog populations [39],[40].

To eliminate infection, disease control efforts need to be targeted at the maintenance population [41]. This is clearly demonstrated for fox rabies in Western Europe, whereby control of rabies in foxes (through mass oral vaccination) has led to the disappearance of rabies from all other ‘spill-over’ hosts [42]. Despite the predominance of domestic dog rabies in Africa, the role of wildlife as independent maintenance hosts has been debated, and many perceive the abundance of wildlife as a barrier to elimination of canine rabies on the continent. It has also been argued that the predominance of dog rabies is an artefact of poor surveillance and under-reporting in wildlife populations [43].

In the wildlife-rich Serengeti ecosystem in Tanzania, evidence suggests that domestic dogs are the only population essential for maintenance [10],[13],[16]: (1) phylogenetic data showed only a single southern Africa canid-associated variant (Africa 1b) circulating among different hosts [16]; (2) transmission networks suggested that, for wildlife hosts, within-species transmission cannot be sustained [16]; and (3) statistical inference indicated that cross-species transmission events from domestic dogs resulted in only relatively short-lived chains of transmission in wildlife with no evidence for persistence [10]. The conclusion that domestic dogs are the only maintenance population in such a species-rich community suggests that elimination of canine rabies through domestic dog vaccination is a realistic possibility, and provides grounds for optimism for wider-scale elimination efforts in Africa. In other parts of central and west Africa, transmission of rabies appears to be driven by domestic dogs [44]. An outstanding question relates to southern Africa. Earlier and recent evidence indicate that jackal species (*Canis mesomelas* and *C. adustus*) and bat-eared foxes (*Otocyon megalotis*) may maintain the canid variant in specific geographic loci in South Africa and Zimbabwe [2], [36]–[38], [45]–[50], but it is still not clear whether these cycles can be sustained over large spatial and temporal scales in the absence of dog rabies [13],[51],[52]. Independent wildlife cycles may preclude continent-wide elimination of this variant through dog vaccination alone and wildlife rabies control strategies, in conjunction with dog vaccination, may need to be considered in specific locations [38].

A critical proportion of the population must be protected (P_crit_) to eliminate infection and this threshold can be calculated from the basic reproductive number (R_0_, defined as the average number of secondary infections caused by an infected individual in a susceptible population) [53]. Vaccinating a large enough proportion of the population to exceed P_crit_ will not only protect the vaccinated individuals but will reduce transmission such that, on average, less than one secondary infection will result from each primary case (effective reproductive number, R_e_<1), which can ultimately lead to elimination. Vaccination has eliminated canine rabies in many countries demonstrating the success of this concept [54]. However, theory suggests that R_0_ increases with population density [39] and thus higher coverage will be needed in higher density populations. However, evaluation of historical outbreak data from around the world and recent data from Tanzania indicate that R_0_ in domestic dog populations is consistently low (between 1.0 and 2.0) [12], confirming the feasibility of rabies elimination through vaccination in African domestic dog populations.

An important conclusion of this study was that in populations with rapid turnover (such as those in many African countries) at least 60% of the population must be vaccinated during annual campaigns to prevent coverage falling below P_crit_ between campaigns. Data from Africa clearly show that very few control efforts have reached these levels of coverage [Table 2], which is why rabies remains a persistent problem [12]. Although emergence of new variants maintained in wildlife also remains a possibility, as shown in the USA, where wildlife rabies now dominates since elimination of canine rabies [55]. For Africa, these questions are likely only to be resolved with large-scale intervention involving mass vaccination of dogs.

**Table 2:** Reported and estimated vaccination coverages in domestic dog populations from various settings in sub-Saharan Africa since 1990.

Region	Country	Dates	Vaccines delivered	Dog population	Estimated coverage (%)	Source of data and notes
N'djamena	Chad	2001	23,560		19.00	XLV
Machakos	Kenya	1992			24.00	XIV
National	Kenya	2003			33.00	XXXIX
Mzuzu	Malawi	1996–2000	7823*	44,932	12.1–20.2	XLVII
National	Mozambique	1997–2000	175,769*	7,000,000	<1	XLVIII
Northern communal land	Namibia	2001		115,000	12.00	XXIX
Borno State (urban)	Nigeria	2007			<46.00	XLIX
Borno State (rural)	Nigeria	2007			<15.6	XLIX
National	Sudan	1992–2002	37,620*	71,540	5.26	Dog population from 1992 census data reported in XXXVI
Khartoum state	Sudan	2000	2,946	91,000	3.24	L
National	Swaziland	1994–1998	57,204		63.2–91.7 (dropped to 3% in 1998)	LI
National	Tanzania	1992	11,635		<1	Extrapolated from LII using human:dog ratios from XLVI, census data from LIII and estimated dog population growth rates from LIV
National	Uganda	2001–2003			16.00	XXVI
National	Zimbabwe	2002	314,319	1,300,000	13.93	Extrapolated from LV, with dog population sizes and growth rates in 1986 from LVI

Targeted mass vaccination campaigns carried out by research projects have been excluded (e.g. XLIV-XLVI).

*Indicates total vaccinations delivered over stated period. See Appendix S1 for references.

### (c) Operational constraints

Several arguments are given for why mass vaccination campaigns have failed to achieve the high levels of coverage that are necessary to interrupt rabies transmission. We counter these arguments below:

#### A perception of many inaccessible stray/ownerless dogs

A common claim is that the majority of dogs in Africa are unowned ‘stray’ animals, and therefore inaccessible for parenteral vaccination. It is not hard to see why this perception has arisen - unrestrained dogs, without any apparent evidence of ownership, are commonly observed. Further investigation, however, usually reveals that the vast majority are owned, and at least one household claims some responsibility, including presentation for vaccination. Published studies in Africa, which quantify the proportion of unowned dogs, are admittedly sparse, but all support this observation [56]–[58]. Capture-mark-recapture methodologies and household questionnaires used in African settings have all found consistently low estimates (Tunisia ∼7% [57], 1%, 8% and 11% in three sites in N'Djamena, Chad [56], and 1% in a peri-urban site in Tanzania [58]). Notably, the Tanzanian site was selected specifically on the basis of reports of many unowned dogs. While mark-recapture methods yield reliable estimates of unowned dog numbers, their implementation and analysis is not trivial and efforts are underway to develop simpler, yet robust methodologies [59]. Certainly in traditional Africa, i.e. most of sub-Saharan Africa, the issue of roaming dogs seems not to be one of a lack of ownership, but rather an inability or unwillingness by owners to confine their dogs.

#### Unwillingness/inability to bring dogs for vaccination

Published studies tend to refute the idea that owners are often unable or unwilling to restrain their dogs for parenteral vaccination. A multi-country WHO-commissioned study (Tunisia, Sri Lanka and Ecuador) concluded that “dogs which are not catchable by at least one person are rare and represent generally less than 15% of the dog population” [57]. Similarly a study from Nepal found that 86–97% of dogs were accessible to parenteral vaccination [60]. Although an early study in Turkey concluded that 48% of all free-roaming owned dogs could not be captured by their owners [61], more recent surveys found that most unvaccinated dogs could be handled (only 16% could not) and that a much larger proportion (56%) resulted from a lack of information about the campaign – a much easier problem to remedy (unpublished data). In Africa, very similar figures were obtained in a multi-site study in urban and rural Tanzania, where only 15% of vaccination failures were due to a reported inability by the owner to handle the dog, while 53% of cases were due to poor information dissemination [17]. However, there may be settings in transitional Africa (e.g. parts of southern Africa including KwaZulu Natal [2]) where handling of dogs is more difficult due to a break-down in traditional animal husbandry and other social factors, and more intensive efforts may be required for these special cases.

Given that most dogs are accessible for parenteral vaccination, high coverage can be achieved with well-planned vaccination campaigns. During pilot programmes in urban and rural Africa which have not charged owners for vaccination, coverages obtained have exceeded 60% [14],[17],[56]. Pastoral communities pose particular challenges due to remote locations and semi-nomadic lifestyles, but >80% coverages can still be achieved through house-to-house delivery strategies or community-based animal health workers [17].

Young pups usually make up a large proportion (>30%) of African dog populations [62] and there is a widespread perception among veterinary authorities and dog owners that they should not be vaccinated, which leads to insufficient coverage [17]. However, rabies vaccines can safely be administered to pups <3 months of age [63], and in village campaigns in Tanzania, vaccines consistently induced high levels (>0.5 IU/ml) of rabies virus neutralizing antibody [64]. The issue of inclusion of pups can effectively be addressed through appropriate advertising before campaigns.

Cost-recovery, through charging dog owners for rabies vaccination, is widely promoted for sustainable programmes and to encourage responsible dog ownership. However, charging for a vaccination that represents a public rather than a private good, can be counterproductive, resulting in low turnouts and coverage (<30%) with little or no impact [65]. Charging for vaccination may indeed be the principal reason why owners are unwilling to bring dogs for vaccination.

Ineffective campaigns that achieve <30% coverage are a waste of resources and can be highly demoralising for veterinary staff and communities. When resources are spread thinly, such that only low coverage is achieved or only small pockets are well vaccinated, then large-scale failure is inevitable. A more epidemiologically sensible strategy is to focus resources into a single (preferably well-bounded) area where high coverage can be consistently achieved.

#### Uncertainty about dog population sizes and ecology for effective design and planning of vaccination campaigns

**Figure 3:**
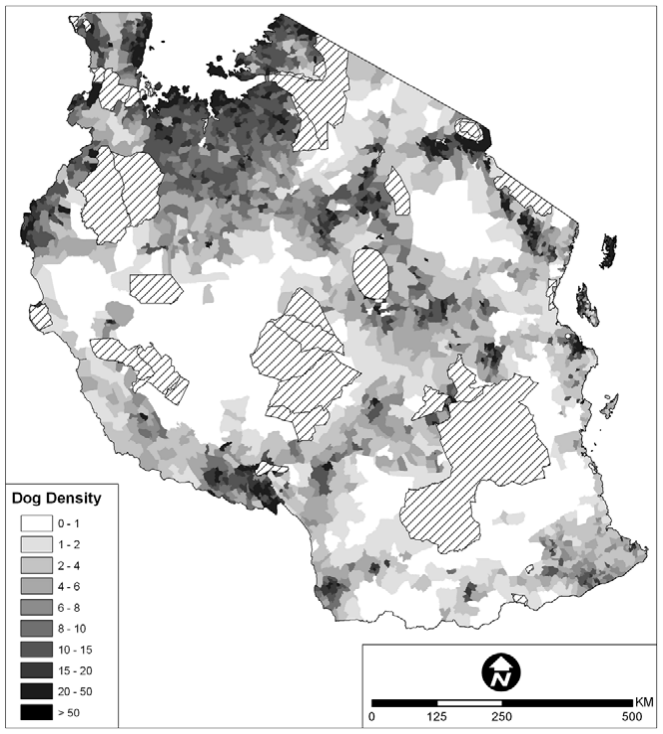
‘Dog density’ map of Tanzania (courtesy of Hawthorne Beyer; data source: LXV in Appendix S1). Hashed areas represent the location of wildlife protected areas.

Official figures used for planning frequently underestimate true population sizes. For example, Gsell [58] found that the owned dog population in a municipality in Tanzania was six times larger than official records. Although standard survey methodologies for estimating dogs/household or dog∶human ratios [57], [66]–[68] are not without problems (for example, double ownership of dogs), a rough estimate of owned dog populations can be derived from national (human) population censuses, and can be corrected for different demographic and ecological settings [18],[69]. More detailed studies can be conducted to identify key household determinants of dog ownership (for example, religion, age and sex of household heads, household size, socio-economic level, and livestock presence/absence [18],[28],[70],[71]. Such determinants have been used to generate a ‘dog density’ map of Tanzania, for assistance in planning national rabies vaccination campaigns (Figure 3).

### (d) Lack of resources

The above factors are all generally described as obstacles that ultimately lead to a lack of investment into rabies control and surveillance. We suggest that investment would actually reap multiple benefits including economic ones, if appropriate strategies are implemented overcoming the constraints described.

#### A lack of surveillance and diagnostic capacity for rabies detection

Poor surveillance and diagnosis capacity means that (1) data is insufficient to demonstrate disease burden and motivate policy-makers, and (2) impacts of control efforts cannot be evaluated.

Considerable progress has been made in the development of simple and inexpensive techniques for sample preservation and rapid post-mortem diagnosis suitable for laboratories with limited storage and/or diagnostic resources with potential to increase in-country capabilities for surveillance. A new direct rapid immunohistochemical test (dRIT) requires only light microscopes [72], which are widely available. The test is simple and can be performed by a range of operators if appropriate training is provided. Field evaluation studies in Africa demonstrated that this assay has characteristics equivalent to those of the direct fluorescent antibody (DFA) test, the global standard for rabies diagnosis, including excellent performance on glycerolated field brain material [15],[73], the preservative of choice under field conditions [74],[75]. Other simple field-diagnostics that allow rapid screening, including enzyme immunoassays [76], dot blot enzyme immunoassays [77] and lateral-flow immunodiagnostic test kits [78],[79] are being evaluated. These tools offer hope of extending diagnostic capacity in resource-limited settings.

Animal-bite injury data from hospitals are an easily accessible source of epidemiological information and have been verified as reliable indicators of animal rabies incidence and human exposures [11],[14]. Furthermore, increasing availability of communication infrastructure through mobile phone network access in remote areas could enhance surveillance by allowing real-time reporting.

#### Costs of effective dog vaccination campaigns are beyond the budget of veterinary services

Veterinary services in Africa usually report very limited budgets and often have to divert resources during outbreaks of other diseases [80],[81]. This is clearly the most significant constraint to effective rabies control. However, with increasing human and dog populations, dog rabies incidence, human exposures to rabies and the costs required to prevent human rabies deaths through PEP will invariably continue to rise unless rabies can be controlled at the source, i.e. in domestic dog populations [82]. Many countries in Asia, such as Thailand, Vietnam and Sri Lanka have greatly reduced human rabies deaths through increased PEP use, but at a very high cost [83]. In Vietnam, for example, deaths fell from 285 in 1996 to 82 in 2006 with administration of >600,000 PEP courses per year at an estimated cost of ∼$27 million/year [84].

**Figure 4:**
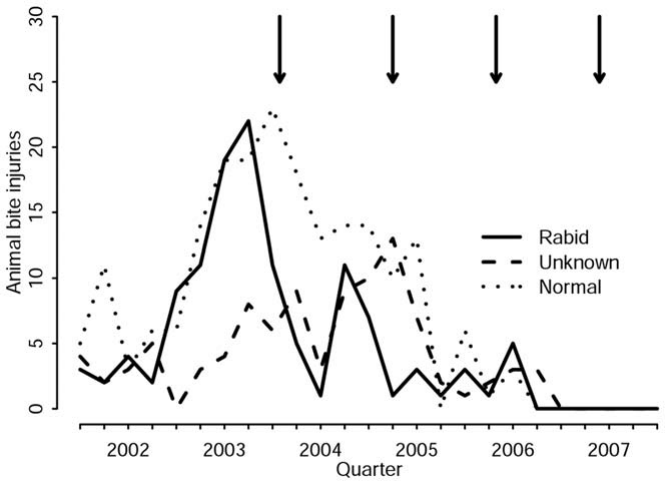
Number of cases of bite injuries reported to hospitals in pastoralist communities to the east of Serengeti National Park (north-western Tanzania). Numbers are recorded as a result of bites from both rabid and normal healthy animals as well as those of unknown status (either the bite victims could not be traced, or insufficient information could be obtained during interviews to make an informed judgement about the health of the biting animal). The arrows mark the end of successive dog vaccination campaigns.

Although domestic dog populations need to be targeted for the effective control of rabies, this is usually deemed to be the responsibility of veterinary services even though many of the benefits accrue to the medical sector. In rural Tanzania, dog vaccination campaigns led to a rapid and dramatic decline in demand for costly human PEP [14]. In pastoral communities, vaccination not only reduced rabies incidence, but has now resulted in a complete absence of exposures reported in local hospitals for over two years (Figure 4).

Large-scale campaigns can therefore translate into human lives and economic savings through reduced demand for PEP. Costs per dog vaccinated are generally estimated to be low (rural Tanzania ∼$1.73 [17], Philippines ∼$1.19–4.27 [85], Tunisia ∼$1.3 [86], Thailand ∼$1.3 [86] and Urban Chad ∼$1.8 [87]) and preliminary studies suggest that including dog vaccination in human rabies prevention strategies would be a highly cost-effective intervention at ∼US $25/DALY averted (S. Cleaveland, unpublished data; see also 82).

Developing joint financing schemes for rabies prevention and control across medical and veterinary sectors would provide a mechanism to use savings in human PEP to sustain rabies control programs in domestic dogs. Although conceptually simple, the integration of budgets across different Ministries is likely to pose political and administrative challenges. However, given sufficient political will and commitment, developing sustained programmes of dog vaccination that result in canine rabies elimination should be possible.

In conclusion, here we show that a substantial body of epidemiological data have now been gathered through multiple studies demonstrating that: (1) rabies is an important disease that exerts a substantial burden on human and animal health, local and national economies and wildlife conservation, (2) domestic dogs are the sole population responsible for rabies maintenance and main source of infection for humans throughout most of Africa and Asia and therefore control of dog rabies should eliminate the disease, (3) elimination of rabies through domestic dog vaccination is epidemiologically feasible, (4) the vast majority of domestic dog populations across sub-Saharan Africa are accessible for vaccination and the few remaining factors compromising coverage can be addressed by engaging communities through education and awareness programs, (5) new diagnostic and surveillance approaches will help evaluate the impact of interventions and focus efforts towards elimination, and (6) dog rabies control is affordable, but is likely to require intersectoral approaches for sustainable programmes that will be needed to establish rabies-free areas.

## Supporting Information

Appendix S1 Appendix with additional references.
(0.07 MB DOC)
