# Supplementary material for: The Feasibility of Canine Rabies Elimination in Africa: Dispelling Doubts with Data
Source: PLoS Negl Trop Dis. 2010 Feb 23;4(2):e626. doi: 10.1371/journal.pntd.0000626 (PMC2826407; doi:10.1371/journal.pntd.0000626)
Supplement: Appendix S1 — Appendix with additional references. (0.07 MB DOC) [file pntd.0000626.s001.doc]

**Appendix S1 - List of references related to tables and figures**

1. Meslin FX, Fishbein DB, Matter HC (1994) Rationale and prospects for rabies elimination in developing countries. In: Rupprecht CE, Dietzschold B and Koprowski H, editors. *Lyssaviruses*. Berlin: Springer Verlag. pp. 1-26.
2. Murray CJL, Lopez AD (1996) The global burden of disease: comprehensive assessment of mortality and disability from diseases, injuries and risk factors in 1990 and projected to 2020. Boston (MA): Harvard University Press.
3. Cleaveland S. (1998) Royal Society of Tropical Medicine and Hygiene meeting at Manson House, London, 20 March 1997. Epidemiology and control of rabies. The growing problem of rabies in Africa. Trans Roy Soc Trop Med Hyg 92: 131-134.
4. World Health Organization (2002) The world health report 2002: reducing risks, promoting healthy life. Geneva: World Health Organization.
5. Laxminarayan R, Mills AJ, Breman JG, Measham AR, Alleyne G et al. (2006) Advancement of global health: key message from the Disease Control Priorities Project. Lancet 367: 1193 – 1208.
6. Dodet B; Africa Rabies Bureau (AfroREB), Adjogoua EV, Aguemon AR, Amadou OH, Atipo AL, Baba BA et al. (2008) Fighting rabies in Africa: the Africa Rabies Expert Bureau (AfroREB). Vaccine 26: 6295-6298.
7. Swanepoel R, Barnard BJH, Meredith CD, Bishop GC, Brückner GK et al. (1993) Rabies in Southern Africa. Onderstepoort J Vet Res 60: 325-346.
8. Nel LH, Thomson GR, Von Teichman BF (1993) Molecular epidemiology of rabies virus in South Africa. Onderstepoort J Vet Res 60: 301-306.
9. Thomson GR, Meredith CD (1993) Rabies in bat-eared foxes in South-Africa. Onderstepoort J Vet Res 60: 399-403.
10. von Teichman BF, Thomson GR, Meredith CD, Nel LH (1995) Molecular epidemiology of rabies virus in South Africa: evidence for two distinct virus groups. J Gen Virol 76: 73-82.
11. Coleman PG, Dye C (1996) Immunization coverage required to prevent outbreaks of dog rabies. Vaccine 14: 185-186.
12. Bingham J, Foggin CM, Wandeler AI, Hill FWG (1999) The epidemiology of rabies in Zimbabwe. 2. Rabies in jackals (*Canis adustus and Canis mesomelas*). Onderstepoort J Vet Res 66: 11-23.
13. East ML, Hofer H, Cox JH, Wulle U, Wiik H et al. (2001) Regular exposure to rabies virus and lack of symptomatic disease in Serengeti spotted hyenas. Proc Natl Acad Sci USA 98: 15026-15031.
14. Kitala PM, McDermott JJ, Kyule MN, Gathuma JM, Perry BD et al. (2001) Dog ecology and demographic information to support planning of rabies control in Machakos District, Kenya. Acta Trop 78: 217-230.
15. Kitala PM, McDermott JJ, Coleman PG, Dye C (2002) Comparison of vaccination strategies for the control of dog rabies in Machakos District, Kenya. Epidemiol Infect 129: 215-222.
16. Sabeta CT, Bingham J, Nel LH (2003) Molecular epidemiology of canid rabies in Zimbabwe and South Africa. Virus Res 91: 203-211.
17. Nel LH, Sabeta CT, von Teichman B, Jaftha JB, Rupprecht CE et al. (2005) Mongoose rabies in southern Africa: a re-evaluation based on molecular epidemiology. Virus Res 109: 165-173.
18. Bingham J (2005) Canine rabies ecology in Southern Africa. Emerg Infect Dis 11: 1337-1342.
19. Coetzee P, Nel LH (2007) Emerging epidemic dog rabies in coastal South Africa: a molecular epidemiological analysis. Virus Res 126: 186-195.
20. Macharia MJ, Kasiiti JL, Karuga AK, Mburu JW, Gacheru SG (2001) Rabies in Kenya. Proceedings of the sixth Southern and Eastern African Rabies Group/World Health Organization meeting, Lilongwe, Malawi: 40-44.
21. Mettler FM, Uanguta M and Hübschle OJB (2001) Control of Rabies in Namibia. Proceedings of the sixth Southern and Eastern African Rabies Group/World Health Organization meeting, Lilongwe, Malawi: 38-39.
22. Rutebarika CS (2001) Rabies in Uganda. Proceedings of the sixth Southern and Eastern African Rabies Group/World Health Organization meeting, Lilongwe, Malawi: 55-59.
23. Awahndukum J, Tchoumboue J, Tong JC (2002) Canine and human rabies in Cameroon. Trop Vet 20: 162-168.
24. Lukhele S (2003) Opening Speech. Proceedings of the seventh Southern and Eastern African Rabies Group/World Health Organization meeting, Ezulwini, Swaziland: 15-17.
25. Liebenberg A (2003) Rabies in South Africa. Proceedings of the seventh Southern and Eastern African Rabies Group/World Health Organization meeting, Ezulwini, Swaziland: 51-54.
26. Rutebarika CS (2003) Rabies in Uganda. Proceedings of the seventh Southern and Eastern African Rabies Group/World Health Organization meeting, Ezulwini, Swaziland: 59-64.
27. Hassan Ali Y (2003) Rabies in Sudan. Proceedings of the seventh Southern and Eastern African Rabies Group/World Health Organization meeting, Ezulwini, Swaziland: 55-57.
28. Rupprecht CE, Barrett J, Briggs D, Cliquet F, Fooks AR et al. (2008) Can rabies be eradicated? Dev Biol (Basel) 131: 95-122.
29. Mettler F (2003) Some aspects of the rabies epizootic in Namibia. Proceedings of the seventh Southern and Eastern African Rabies Group/World Health Organization meeting, Ezulwini, Swaziland: 43-46.
30. Tekleghiorghis T and Yosief T (2003) Rabies in Eritrea. Proceedings of the seventh Southern and Eastern African Rabies Group/World Health Organization meeting, Ezulwini, Swaziland: 27-29.
31. Mangulama LK (2001) Opening speech. Proceedings of the sixth Southern and Eastern African Rabies Group/World Health Organization meeting, Lilongwe, Malawi: 10-11.
32. Cleaveland S, Kaare M, Tiringa P, Mlengeya T (2001) A dog rabies vaccination campaign in rural Africa: Impact on the incidence of animal rabies and human bite injuries. Proceedings of the sixth Southern and Eastern African Rabies Group/World Health Organization meeting, Lilongwe, Malawi: 114-126.
33. Wandeler AI (2001) Some observations on rabies epidemiology and control. Proceedings of the sixth Southern and Eastern African Rabies Group/World Health Organization meeting, Lilongwe, Malawi: 166-170.
34. de Balogh KKIM, Frumau EH, Hankanga C (2001) Perception and knowledge about rabies. Proceedings of the sixth Southern and Eastern African Rabies Group/World Health Organization meeting, Lilongwe, Malawi: 186-191.
35. Weyer J, Viljoen GJ, Nel LH (2003) Alternative recombinant poxvirus vaccine for rabies. Proceedings of the seventh Southern and Eastern African Rabies Group/World Health Organization meeting, Ezulwini, Swaziland: 79-84.
36. Ali YH, Intisar KS, Wegdan HA, Ali EB (2006) Epidemiology of rabies in Sudan. J Anim Vet Adv 5: 266-270.
37. Zinsstag J, Dürr S, Penny MA, Mindekem R, Roth F et al. (2009) Transmission dynamics and economics of rabies control in dogs and humans in an African city. Proc Natl Acad Sci USA 106: 14996-15001.
38. Markotter W, York D, Sabeta CT, Shumba W, Zulu G et al. (2009) Evaluation of a rapid immunodiagnostic test kit for detection of African lyssaviruses from brain material. Onderstepoort J Vet Res 76: 257-262.
39. Macharia MJ, Ombacho KM, Kasiiti JL, Mbugua HCW, Gacheru SG (2003) Status of rabies in Kenya 1998 – 2002. Proceedings of the seventh Southern and Eastern African Rabies Group/World Health Organization meeting, Ezulwini, Swaziland: 31-38.
40. Vos A, Aylan O, Estrada R (2003) Oral vaccination campaigns of dogs against rabies. Proceedings of the seventh Southern and Eastern African Rabies Group/World Health Organization meeting, Ezulwini, Swaziland: 125-130.
41. Swanepoel R (1994) Rabies. In Coetzer JAW, Thomson GR, Tustin RC, editors. Infectious diseases of livestock with special reference to Southern Africa. pp. 493-552.
42. Proceedings of the sixth Southern and Eastern African Rabies Group/World Health Organization meeting, Lilongwe, Malawi, 2001.
43. Proceedings of the seventh Southern and Eastern African Rabies Group/World Health Organization meeting, Ezulwini, Swaziland, 2003.
44. Cleaveland S, Kaare M, Tiringa P, Mlengeya T, Barrat J (2003) A dog rabies vaccination campaign in rural Africa: impact on the incidence of dog rabies and human dog-bite injuries. Vaccine 21: 1965-1973.
45. Kayali U, Mindekem R, Yémadji N, Vounatsou P, Kaninga Y et al. (2003) Coverage of pilot parenteral vaccination campaign against canine rabies in N'Djaména, Chad. Bull World Health Organ 81: 739-744.
46. Kaare M, Lembo T, Hampson K, Ernest E, Estes A et al. (2009) Rabies control in rural Africa: evaluating strategies for effective domestic dog vaccination. Vaccine 27: 152-160.
47. Chimera BAR and Chikungwa PB (2001) Rabies in Malawi. Proceedings of the sixth Southern and Eastern African Rabies Group/World Health Organization meeting, Lilongwe, Malawi: 30-33.
48. Pinto ME (2001) Rabies in Mozambique. Proceedings of the sixth Southern and Eastern African Rabies Group/World Health Organization meeting, Lilongwe, Malawi: 34-37.
49. El-Yuguda AD, Baba AA, Baba SSA (2007) Dog population structure and cases of rabies among dog bite victims in urban and rural areas of Borno State, Nigeria. Trop Vet 25: 34-40.
50. Ali YH (2001) Rabies in Sudan. Proceedings of the sixth Southern and Eastern African Rabies Group/World Health Organization meeting, Lilongwe, Malawi: 50-52.
51. Dlamini RX (1999) Rabies in Swaziland. Proceedings of the Southern and Eastern African Rabies Group, Entebbe, Uganda: 34-36.
52. Sembiko YSS (1995) Rabies in Tanzania. Proceedings of the third International Conference of the Southern and Eastern African Rabies Group, Harare, Zimbabwe: 29-32.
53. Tanzania National Bureau of Statistics. 2005. 2002 Population and housing census. President's Office, Planning and Privatization, Dar es Salaam.
54. Hampson K, Dushoff J, Cleaveland S, Haydon DT, Kaare M et al. (2009) Transmission dynamics and prospects for the elimination of canine rabies. PLoS Biol 7: e53.
55. Shumba W (2003) Rabies in Zimbabwe 1999 – 2002. Proceedings of the seventh Southern and Eastern African Rabies Group/World Health Organization meeting, Ezulwini, Swaziland: 65-68.
56. Brooks R (1990) Survey of the dog population of Zimbabwe and its level of rabies vaccination. Vet Rec 127: 592-596.
57. Knobel DL, Cleaveland S, Coleman PG, Fèvre EM, Meltzer MI et al. (2005) Re-evaluating the burden of rabies in Africa and Asia. Bull World Health Organ 83: 360-368.
58. World Health Organization (2004) Disease and injury regional estimates for 2004. Available at: <http://www.who.int/healthinfo/global_burden_disease/estimates_regional/en/index.html. Accessed 23/3/09>.
59. World Health Organization (2003) Cumulative number of reported probable cases of severe acute respiratory syndrome (SARS), epidemic and pandemic alert and response. Available at: <http://www.who.int/csr/sars/country/table2004_04_21/en/index.html. Accessed 25/3/09>.
60. World Health Organization (2008) Cumulative number of confirmed human cases of avian influenza A/(H5N1) reported to WHO. Available at: <http://www.who.int/csr/disease/avian_influenza/country/cases_table_2008_02_21/en/index.html. Accessed 23/3/09>.
61. World Health Organization (2001) Nipah virus, Fact Sheets. Available at: <http://www.who.int/mediacentre/factsheets/fs262/en/>
62. World Health Organization (2007) Rift Valley Fever in Kenya, Somalia and the United Republic of Tanzania. Disease Outbreak News, 9 May 2007, <http://www.who.int/csr/don/2007_05_09/en/index.html>
63. World Health Organization (2008) Rift Valley Fever in Sudan. Disease Outbreak News, 22 January 2008, <http://www.who.int/csr/don/2008_01_22/en/index.html>
64. Coleman PG, Fèvre EM, Cleaveland S (2004) Estimating the public health impact of rabies. Emerg Infect Dis 10: 140-142.
65. Knobel DL (2009) Aspects of dog ownership and canine rabies control in Africa and Asia. PhD thesis, University of Edinburgh.
